# Time Series Genome-Centric Analysis Unveils Bacterial Response to Operational Disturbance in Activated Sludge

**DOI:** 10.1128/mSystems.00169-19

**Published:** 2019-07-02

**Authors:** María Victoria Pérez, Leandro D. Guerrero, Esteban Orellana, Eva L. Figuerola, Leonardo Erijman

**Affiliations:** a Instituto de Investigaciones en Ingeniería Genética y Biología Molecular “Dr. Héctor N. Torres” (INGEBI-CONICET) Vuelta de Obligado, Buenos Aires, Argentina; b Agua y Saneamientos Argentinos S. A. Tucumán, Buenos Aires, Argentina; c Departamento de Fisiología, Biología Molecular y Celular, Facultad de Ciencias Exactas y Naturales, Universidad de Buenos Aires, Buenos Aires, Argentina; Queen's University Belfast

**Keywords:** genome-centric metagenomics, activated sludge, metagenomics, replication index, *rrn* operon copy number, time series, wastewater treatment

## Abstract

Understanding ecosystem response to disturbances and identifying the most critical traits for the maintenance of ecosystem functioning are important goals for microbial community ecology. In this study, we used 16S rRNA amplicon sequencing and metagenomics to investigate the assembly of bacterial populations in a full-scale municipal activated sludge wastewater treatment plant over a period of 3 years, including a 9-month period of disturbance characterized by short-term plant shutdowns. Following the reconstruction of 173 metagenome-assembled genomes, we assessed the functional potential, the number of rRNA gene operons, and the *in situ* growth rate of microorganisms present throughout the time series. Operational disturbances caused a significant decrease in bacteria with a single copy of the rRNA (*rrn*) operon. Despite moderate differences in resource availability, replication rates were distributed uniformly throughout time, with no differences between disturbed and stable periods. We suggest that the length of the growth lag phase, rather than the growth rate, is the primary driver of selection under disturbed conditions. Thus, the system could maintain its function in the face of disturbance by recruiting bacteria with the capacity to rapidly resume growth under unsteady operating conditions.

**IMPORTANCE** Disturbance is a key determinant of community assembly and dynamics in natural and engineered ecosystems. Microbiome response to disturbance is thought to be influenced by bacterial growth traits and life history strategies. In this time series observational study, the response to disturbance of microbial communities in a full-scale activated sludge wastewater treatment plant was assessed by computing specific cellular traits of genomes retrieved from metagenomes. It was found that the genomes observed in disturbed periods have more copies of the rRNA operon than genomes observed in stable periods, whereas the *in situ* mean relative growth rates of bacteria present during stable and disturbed periods were indistinguishable. From these intriguing observations, we infer that the length of the lag phase might be a growth trait that affects the microbial response to disturbance. Further exploration of this hypothesis could contribute to better understanding of the adaptive response of microbiomes to unsteady environmental conditions.

## Abstract (precis)

Disturbance is a key determinant of community assembly and dynamics in natural and engineered ecosystems. Microbiome response to disturbance is thought to be influenced by bacterial growth traits and life history strategies. In this time series observational study, the response to disturbance of microbial communities in a full-scale activated sludge wastewater treatment plant was assessed by computing specific cellular traits of genomes retrieved from metagenomes. It was found that the genomes observed in disturbed periods have more copies of the rRNA operon than genomes observed in stable periods, whereas the *in situ* mean relative growth rates of bacteria present during stable and disturbed periods were indistinguishable. From these intriguing observations, we infer that the length of the lag phase might be a growth trait that affects the microbial response to disturbance. Further exploration of this hypothesis could contribute to better understanding of the adaptive response of microbiomes to unsteady environmental conditions.

## INTRODUCTION

Understanding the drivers of community structure is important for developing predictive models and for guiding engineering and management practices of microbial community ecosystems (1–4). By contributing to environmental heterogeneity, disturbance is a particularly important driver in shaping species composition in many ecosystems (5). Yet, although it has been the focus of ecological research for a very long time, community response to disturbance remains difficult to predict (6), especially for microbial communities (7). After surveying a few hundreds of studies of soil, marine and freshwater, engineered, and host-associated systems, Shade and colleagues concluded that the majority of microbial communities were sensitive to pulse disturbances in either composition, function, or both (8). In most cases, altered communities recovered function more frequently than composition (8). The maintenance of ecosystem function despite changes in community composition can be largely attributed to the high metabolic flexibility and functional redundancy of microbial systems (9, 10). The ability to recover and return to the original function and composition (i.e., the resilience) is also a feature of many microbiomes (11, 12), due in part to the potential of high growth rates of microorganisms, (7). In addition, species-specific trade-offs between growth and disturbance tolerance may influence the response of microbial communities to disturbance (13, 14). It is then important to understand which traits are the most critical to the maintenance of functioning under disturbed conditions for incorporation into predictive ecosystem models (15–17). Currently, however, there is a gap in the knowledge of microbial traits associated with the response to disturbances. Identification of traits that explain the ability of species to respond to disturbance may also allow for greater insight into the microbial ecology of processes beyond the description of community composition and diversity (18).

Microbial communities in wastewater treatment plants (WWTP) can serve as good model systems to investigate this question, as they are typically able to perform reliably under fluctuating conditions. Although full-scale systems are often subjected to perturbations that may not be well defined, an advantage of field studies is that information can be obtained about the ability of microbial communities to cope with a fluctuating environment in a real scenario. A great deal of knowledge has been achieved during the last 2 decades from research exploring how bacterial community composition is affected by process configuration, solid retention time (SRT), temperature, redox conditions, wastewater composition, pH, and other environmental and operational pressures (19–27). Recent progress in high-throughput sequencing technology has facilitated a detailed characterization of the composition of microbial communities in a large number of wastewater treatment systems worldwide (23, 28–30). However, few studies have addressed the response of microbial communities to disturbance in full-scale systems (31). Vuono et al. observed that a sudden decrease in SRT from 12 to 3 days prompted a shift in community structure, favoring fast-growing organisms that are adapted for high resource availability (22).

Activated sludge systems operate at loading rates typically in the range of 0.05 to 0.4 kg BOD (biochemical oxygen demand) per kg dry biomass and per day and can be therefore considered oligotrophic environments (32). In other words, under stable operation bacteria must adapt to grow under conditions of carbon limitation (22, 33). Indeed, it has been inferred on the basis of the average number of *rrn* operons that activated sludge is dominated by bacterial populations that are near carrying capacity and make efficient use of available growth-limiting resources (22, 34). Thus, ecological selection might be acting at the levels of both metabolic functions and life history strategies of bacterial populations. This hypothesis could be tested directly by examining signatures in the genomes of the bacterial community. Metagenomics offers the possibility to capture the genomic complexity of bacterial communities at high resolution. In particular, genome-centric approaches have the additional advantage that specific traits can be analyzed in the context of the other functional properties of the microorganisms (35).

In this study, we used metagenomics and 16S rRNA sequencing to investigate bacterial population and functional dynamics in a full-scale municipal activated sludge WWTP over a period of 3 years, which comprised distinct operational process conditions. Similar studies have investigated the importance of functional traits for community assembly processes by applying the PICRUSt approach (36) to predict functional profiles of microbial communities using 16S rRNA gene sequences and have estimated the number of rRNA gene operons using the rRNA Database (22, 37). Our analysis differs importantly from those previous studies in that we accomplished genome reconstruction to assess the functional profile, and we estimated the number of rRNA gene operons and the *in situ* growth rate of assembled genomes (38, 39). We first used 16S rRNA amplicon sequencing data to identify patterns of co-occurrence between taxa over the 3-year time series and compared the distribution of community composition with operational and performance metadata, thereby establishing a consensus for periods of disturbance and high stability. Second, we used metagenomic data to identify differences in functional profiles between contrasting operational periods. Finally, we estimated the number of rRNA gene operons and the *in situ* growth rate of reconstructed genomes across the time series that comprised distinctly perturbed and stable periods to test the hypothesis that shifts in bacterial community structure caused by process disturbances reflected differences in functional characteristics of microorganisms, including their growth traits.

## RESULTS

### WWTP performance.

**FIG 1:**
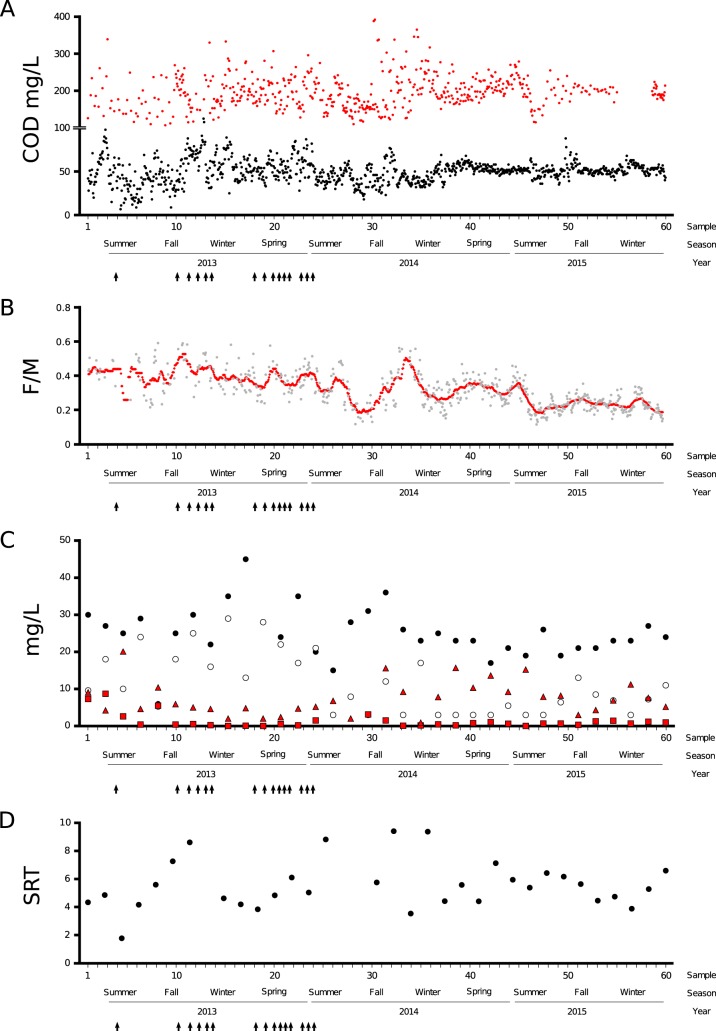
Functional performance and operating conditions of the full-scale activated sludge over 3 years of sampling. (A) Influent (red dots) and effluent (black dots) chemical oxygen demand concentration. (B) Food/microorganism (F/M) ratio. Red dots indicate the F/M calculated using data based on a 28-day moving average (89). (C) Nitrogen profiles over the 3 years of sampling. Black circles, influent total Kjeldahl nitrogen (TKN); white circles, effluent total Kjeldahl nitrogen; red triangles, effluent nitrate concentration; red squares, effluent nitrite concentration. (D) Solid retention time (SRT). The SRT was estimated by dividing the monthly average biomass concentration in the aeration basins under operation by the loss of solids through wastage and effluent during the same period. Black arrows indicate disturbance events.

The main characteristics and average performance data of the municipal full-scale activated sludge plant are given in Table S1 in the supplemental material. The sewage treatment plant was sampled during the course of a planned capacity expansion, which gave rise to a singular period, which lasted approximately 9 months, characterized by repeated disturbances, caused mainly by short-term plant shutdown, each time for several hours. Features of this period were interruption of wastewater feed supply, fluctuations in mixed liquor-suspended solids (MLSS) concentration, and/or eventual periods of very low dissolved oxygen concentration, resulting in higher effluent chemical oxygen demand (COD) (Fig. 1A). After a new operative module was gradually put into operation, there was a moderate decrease in food to microorganism ratio (F/M) (Fig. 1B), which ultimately led to a late period of 9 months of very good performance, which included full nitrification (Fig. 1C). The SRT, ranging between 4 and 7 days, changed in the opposite direction of F/M (Fig. 1D).

10.1128/mSystems.00169-19.8 TABLE S1 Operational parameters and treatment performance of activated sludge wastewater treatment plant. Download Table S1, DOCX file, 0.02 MB. Copyright © 2019 Pérez et al. 2019 Pérez et al. This content is distributed under the terms of the Creative Commons Attribution 4.0 International license.

### Phylogenetic analysis of activated sludge bacterial community structure and dynamics.

**FIG 2:**
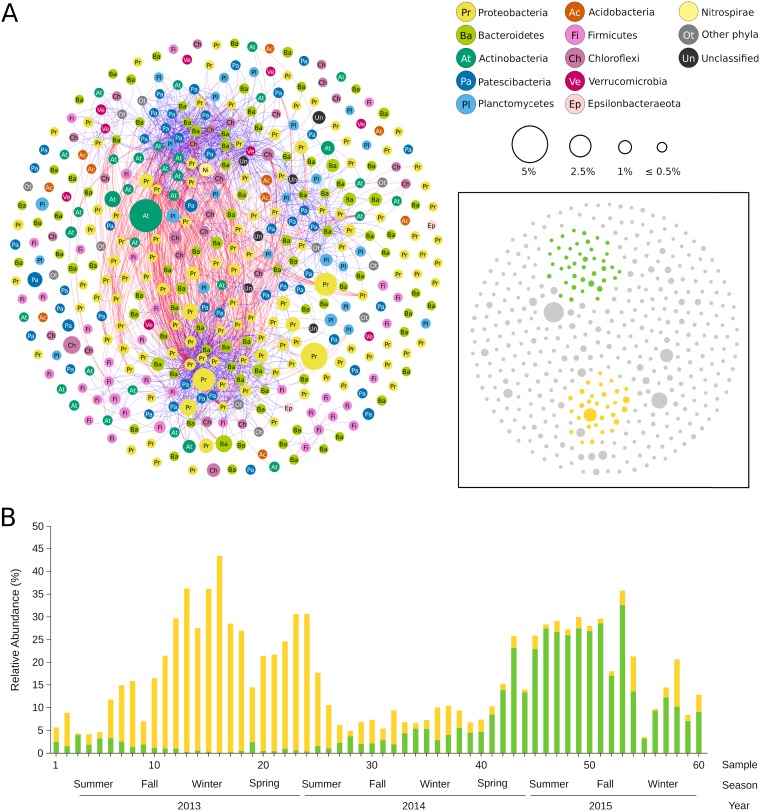
(A) Network of co-occurring bacterial OTUs, based on local similarity analysis (LSA). Nodes (OTUs) are colored by phylum. The size of each node is proportional to the OTU relative abundance across the 60 samples. Blue and red edges represent positive and negative significant correlations, respectively (correlation > |0.6|; *P* < 0.01). Nodes that have no connections are distributed randomly. (Inset) The application of a k-core filter = 10 (considering only positive correlations) highlights two clusters. (Nodes in each cluster are denoted in green and yellow.) (B) Time series abundance of OTUs belonging to the two major clusters of the network shown in panel A. yellow and green bars are the sum of the relative abundance of nodes (OTUs) in each cluster.

Initial analysis of the 3-year time series was based on sequences of 16S rRNA gene amplicons, which were assigned to 1,002 operational taxonomic units (OTUs [97% similarity]) with relative abundance higher than 0.01%. We performed local similarity analysis (LSA) on the most abundant bacterial OTUs in order to explore co-occurrence patterns. The network analysis grouped bacterial OTUs into two main clusters, which were negatively correlated with each other (Fig. 2A). OTUs corresponding to one of the main clusters belong mostly to phyla *Bacteroidetes* and *Proteobacteria*, whereas phyla *Acidobacteria*, *Actinobacteria*, *Patescibacteria*, *Chloroflexi*, *Planctomycetes*, and *Nitrospira* were mostly represented in the other major cluster. A plot of the temporal distribution of bacteria belonging to the two main clusters of the network shows a striking correspondence to each of the two operationally distinct periods of disturbance and stability (Fig. 2B).

Further taxonomic analysis was performed on draft genomes assembled from metagenomes. We were able to reconstruct 173 good-quality metagenome-assembled genomes (MAGs), taking advantage of the differences in contig coverage across the large number of metagenomic data sets (*n* = 60). Table S2 in the supplemental material details the characteristics of all MAGs, including their taxonomic affiliation, length, number of contigs, and degree of completeness and contamination. Similar to the results obtained for amplicons, network analysis of MAGs also showed two distinguishable clusters (see Fig. S1 in the supplemental material).

10.1128/mSystems.00169-19.1 FIG S1 Network of co-occurring MAGs, based on local similarity analysis (LSA). Nodes (MAGs) are colored by phylum. The size of each node is proportional to the average MAG abundance across the 60 samples. Blue and red edges represent positive and negative significant correlations (correlation > |0.6|; *P* < 0.01). Download FIG S1, EPS file, 0.2 MB. Copyright © 2019 Pérez et al. 2019 Pérez et al. This content is distributed under the terms of the Creative Commons Attribution 4.0 International license.

10.1128/mSystems.00169-19.9 TABLE S2 Metagenome-assembled genome (MAG) information. Download Table S2, DOCX file, 0.03 MB. Copyright © 2019 Pérez et al. 2019 Pérez et al. This content is distributed under the terms of the Creative Commons Attribution 4.0 International license.

Given that rRNA genes of microbial communities are in general not efficiently recovered using *de novo* assemblers, we used an iterative mapping method, based on the expectation maximization algorithm, to reconstruct full-length small-subunit (SSU) sequences from raw reads (40). In order to evaluate whether the population sets obtained from the reconstruction of genomes in the metagenomes were a fair representation of the “true” bacterial community structure, we compared the average bacterial community structures determined from (i) the taxonomic classification of 16S rRNA gene amplicons (Amp16S), (ii) the taxonomic annotation of 16S rRNA gene sequences assembled from the metagenomes (MA16S), and (iii) the taxonomic placement of metagenome assembled genomes (MAGs) (see Fig. S2 in the supplemental material).

10.1128/mSystems.00169-19.2 FIG S2 Average relative abundance of bacterial phylum across the 60 samples, determined from the taxonomic placement of metagenome assembled genomes (MAGs), from 16S rRNA gene sequences assembled from metagenomes (MA16S) and from 16S rRNA gene amplicons (Amp16S). Bars indicated standard deviation. Download FIG S2, EPS file, 0.1 MB. Copyright © 2019 Pérez et al. 2019 Pérez et al. This content is distributed under the terms of the Creative Commons Attribution 4.0 International license.

As expected, the distributions of taxa at higher levels were very similar, albeit not identical. The higher sequencing depth afforded by amplicon sequencing allowed resolving for a higher proportion of minor phyla. Additionally, incomplete phylogenetic placement of amplicons resulted in a small proportion of unclassified taxa. Other differences could be explained by the different sources of amplification biases occurring in PCR-based methods, the bias in the estimation of taxa relative abundances due to differences in *rrn* operon copy number, and the incomplete reconstruction of the total community members as MAGs. Despite these differences, the three methods showed a highly satisfactory agreement. Therefore, we conclude that the set of genomes assembled from the metagenomes adequately represent the bacterial communities present in the system, confirming that our sequencing effort provided sufficient resolution to obtain a comprehensive insight in the prokaryotic microbial community of the system.

### Metagenomic analysis.

**FIG 3:**
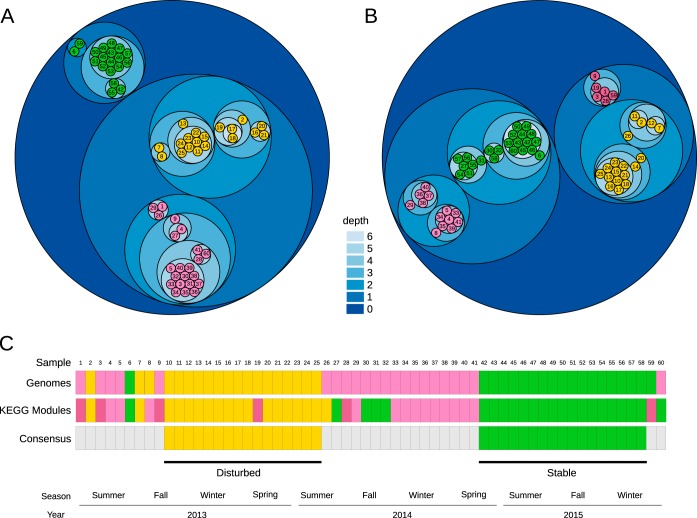
Self-organizing map (SOM) analysis. Shown is a circle packing representation of the tree derived from the 5-by-5 SOM clustering results (Fig. S3). Cluster nodes are visualized as nested circles, with the node levels depicted by a blue gradient. Numbers in childless nodes are those of the time series samples. Samples grouped into superclusters were classified using Ward’s minimum variance method and are displayed in yellow, green, pink, and purple. (A) SOM clustering based on MAG abundance and (B) SOM clustering based on KEGG module abundance. (C) Overlap between samples in superclusters from panels A and B were used to define consensus samples for disturbed (yellow) and stable (green) periods.

The distribution of taxa across the time series was investigated with MAGs as input to construct 5-by-5 self-organizing maps (SOMs) (see Fig. S3 in the supplemental material). SOMs grouped MAGs into two distant superclusters (in green and yellow in Fig. 3A) and a third supercluster (in pink) that includes all other sampling dates. In order to identify differences in functional composition, an analogous SOM analysis was applied to the whole metagenome (Fig. 3B). Relative abundance of annotated genes (KEGG modules) in metagenomic contigs resulted in a similar clustering pattern, with two distant groups of samples (also in green and yellow) and two additional periods (in pink and purple) that include the remaining samples. Comparison between function-based and genome-based SOM analyses enabled us to establish two consensus periods, characterized by their close correspondence to operational periods of disturbance (samples 10 to 25) and stability (samples 42 to 58) (Fig. 3C).

10.1128/mSystems.00169-19.3 FIG S3 Self-organizing map (SOM) analysis. (A) SOM cluster of 60 samples based on MAG abundance and (B) based on KEGG module abundance. Numbers inside boxes (clusters) correspond to samples (1 to 60). Superclusters generated using Ward’s minimum variance method are shown with colors. Download FIG S3, EPS file, 0.09 MB. Copyright © 2019 Pérez et al. 2019 Pérez et al. This content is distributed under the terms of the Creative Commons Attribution 4.0 International license.

Comparison of functional profiles between metagenomes from disturbed and stable periods disclosed a number of pathways that had significant differences in relative abundance (see Fig. S4A and B in the supplemental material). The disturbed period was significantly enriched in genomic features such as secretion system, two-component systems, transporters, and energy metabolism. On the other hand, the stable period contained a significantly higher proportion of genes in the categories of metabolism and genetic information processing and genes coding for biosynthesis of secondary metabolites and genes (Fig. S4B).

10.1128/mSystems.00169-19.4 FIG S4 (A) Relative abundance of KEGG orthology classes showing statistically significant differences between activated sludge samples belonging to periods of disturbance (yellow bars; *n* = 16) and stability (green bars; *n* = 17). Error bars indicate within-group standard deviations. All categories had a corrected *P* value of <0.01 in Welch’s test. (A) KEGG orthology (KO) classifications of metagenomic genes grouped into main classes. (B) KEGG orthology (KO) classifications at the pathway level, including genes grouped into protein families. (C) MAG relative abundance showing statistically significant differences between activated sludge samples belonging to periods of disturbance (yellow bars; *n* = 16) and stability (green bars; *n* = 17). Error bars indicate within-group standard deviations. Shown MAGs passed a corrected *P* value of <0.01 in Welch’s test. Download FIG S4, EPS file, 2.5 MB. Copyright © 2019 Pérez et al. 2019 Pérez et al. This content is distributed under the terms of the Creative Commons Attribution 4.0 International license.

Statistical comparisons were also used to discriminate MAGs according to differences in their abundance between disturbed and stable periods (Fig. S4C). In agreement with the 16S-based results, the abundance of *Proteobacteria* increased during the disturbed period. Nevertheless, most phyla have representatives in both disturbed and stable periods. Likewise, a genome-wide comparison of MAGs, based on predicted metabolic capacities (KEGG modules), indicated that phylogenetically related MAGs, sharing a large number of annotated genes, split into disturbed and stable periods (see Fig. S5 in the supplemental material).

10.1128/mSystems.00169-19.5 FIG S5 Heat map of KEGG modules present in MAGs whose abundance between disturbed and stable periods were significantly different (Fig. S4B). MAGs were colored according to their membership to disturbed (yellow) and stable (green) periods. Download FIG S5, EPS file, 0.7 MB. Copyright © 2019 Pérez et al. 2019 Pérez et al. This content is distributed under the terms of the Creative Commons Attribution 4.0 International license.

### Bacterial community structure and growth traits.

**FIG 4:**
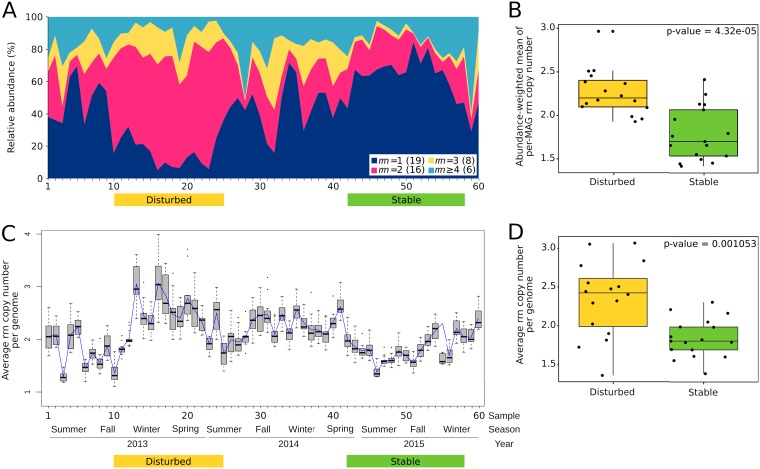
(A) Relative abundance of metagenome-assembled genomes across the 3-year time series, color-coded according to the number of copies of the *rrn* operon. Colored bars delimit samples corresponding to disturbed (yellow) and stable (green) consensus periods defined in Fig. 3. In parentheses are the number of MAGs in each category. (B) Box plot of abundance-weighted mean of per-MAG *rrn* copy number, calculated from all MAGs present in the periods marked as disturbed (yellow) and stable (green). (C) Average copy number of rRNA operons (*rrn*) per genome, calculated as the ratio of the coverage of total rRNA SSU genes to the total coverage of single-copy universal marker genes in metagenomes’ contigs. The box plot was generated using 13 single-copy marker genes. The blue line is the median. (D) Box plot of the average *rrn* copy number per genome in the periods marked as disturbed (yellow) and stable (green).

We searched for traits encoded in bacterial genomes that could account for the differences in bacterial composition observed between disturbed and stable periods. First, we calculated the rRNA (*rrn*) operon copy numbers. We applied an approach for quantifying *rrn* copy numbers of MAGs that is independent of the presence of *rrn* operons in the assembled genomes. Matching MAGs to their corresponding 16S rRNA was initially accomplished by Pearson correlation of the coverage of the MAG and the coverage of the 16S rRNA gene sequences reconstructed independently (MA16S) along the entire time series. The matching was considered robust if the correlation was *r *>* *0.7 (*P* < 0.001) and the taxonomic classifications based on the 16S rRNA sequence and on the genome phylogeny were coincident (see Table S3 in the supplemental material). Further support was obtained from BLAST and/or paired-end (PE) reads connecting the 16S rRNA gene sequences assembled from the metagenomes (MA16S) with contigs in the MAG (see Materials and Methods). The filtering procedure was successfully applied to 49 MAGs, for which we could unequivocally match the reconstructed 16S rRNA gene with the assembled genome. The number of rRNA operons (*rrn*) in each MAG was subsequently calculated from the relationship between the coverage of the MA16S and the average coverage of the rest of the genome (see Fig. S6 and Table S3 in the supplemental material). Results obtained using this approach were further checked by comparing the estimated *rrn* copy number against the copy number retrieved from rrnDB, the rRNA operon copy number database (41) (Table S3). Figure 4A shows that the proportion of genomes with a single copy of *rrn* operon was highest during the period of stable operation and decreased to a minimum during the period of plant disturbance. Differences in the abundance-weighted mean of per-MAG rRNA operon copy number between both contrasting periods were significant at *P* < 0.001 (Fig. 4B). An almost identical result was obtained when the *rrn* copy number was calculated using the contigs directly from metagenomes, as the ratio of the coverage of total rRNA SSU genes (MA16S) to the average coverage of total genomes, determined from the coverage of a set of single-copy marker genes (Fig. 4C and D).

10.1128/mSystems.00169-19.6 FIG S6 Representative illustration of the relationship between MA16 and MAG coverage used to determine the SSU operon copy number. The ID and taxonomic classification of MAGs and MA16S and estimated *rrn* copy number of 8 selected MAGs are indicated in the figure. Lines are absolute coverage values across the 60 samples for MA16S (red lines) and MAGs (blue lines). Numbers of *rrn* copies were calculated as the average of the ratio of coverage between MA16S and MAGs. Only values above a threshold coverage = 1 (dotted line) were used for calculations. Download FIG S6, EPS file, 0.08 MB. Copyright © 2019 Pérez et al. 2019 Pérez et al. This content is distributed under the terms of the Creative Commons Attribution 4.0 International license.

10.1128/mSystems.00169-19.10 TABLE S3 MAG and MA16S data used for the calculation of ribosomal RNA operon copy numbers. Download Table S3, DOCX file, 0.02 MB. Copyright © 2019 Pérez et al. 2019 Pérez et al. This content is distributed under the terms of the Creative Commons Attribution 4.0 International license.

**FIG 5:**
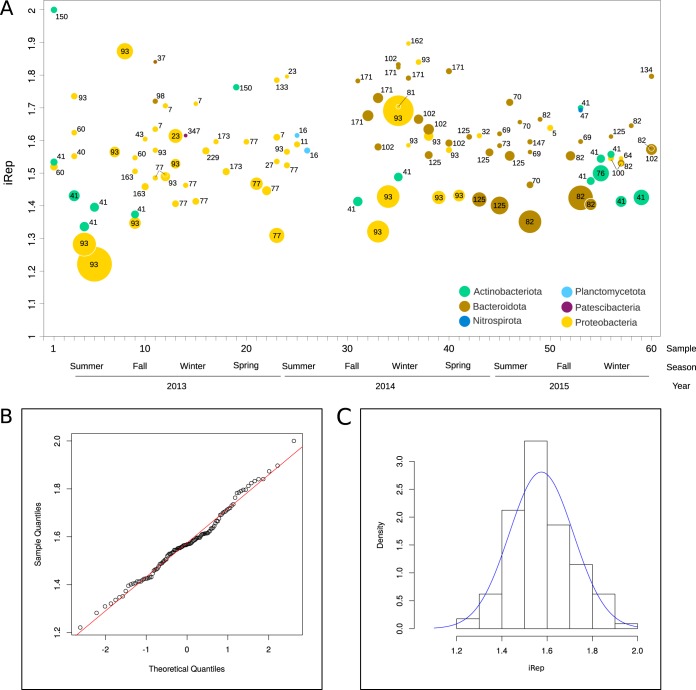
(A) Replication index (iRep) calculated from the difference of coverage between the regions of the genome close to the single replication origin and those of the rest of the metagenome assembled genomes. Values of iRep were calculated for 35 MAGs across 60 samples. Phyla are distinguished by colors. The size of the circles is proportional to the MAG coverage. Numbers denote MAGs according to Table S2. (B) QQ (quantile-quantile) plot of iRep values versus normal distribution. (C) Histogram of iRep values. The solid line in blue represents the normal distribution centered at 1.57 with a standard deviation of 0.14.

Because *rrn* copy number is directly related to maximum growth rate in bacteria (42), we reasoned that differences in *rrn* copy number could be reflected by changes in bacterial growth rate between the disturbed and stable periods. To test this idea, replication rates were estimated across the time series using the index of replication (iRep). The index was estimated for 113 time points in the 35 genomes that were ≥75% complete and had coverage higher than 5 in each sample (Fig. 5A). In contrast to our prediction, iRep values were distributed uniformly throughout time, without differences between disturbed and stable periods, and with no apparent relation to any other measured parameter, including F/M and SRT. However, the lack of association might be a result of low statistical power due to the coarse measurement of SRT (Fig. 1C). The null hypothesis that the distribution of iRep values is indistinguishable from a normal distribution centered at 1.57 with a standard deviation of 0.14 could not be rejected (*P* = 0.39). This was further demonstrated by a very good fit between the quantiles of the iRep data and those of a normal distribution (Fig. 5B and C). Also, we did not find a significant correlation between the copy number of *rrn* operons and iRep values (*n* = 33; Spearman’s correlation *r* = 0.31, *P* = 0.082). Based on these observations, we conclude that bacteria present in disturbed and stable periods are characterized by differences in *rrn* copy number but not by growth rate.

## DISCUSSION

A genome-centric approach using time series metagenomic data from activated sludge samples allowed insight into community response to disturbance. Our results revealed clear differences between disturbed and stable operational periods regarding bacterial rRNA (*rrn*) operon copy numbers. On the other hand, these differences were not reflected in corresponding changes in replication rates.

A stable activated sludge process is a system that produces effluent with very low oxygen demand, thus representing an environment characterized by a constant but slow flux of resources, meaning that bacteria are fed at a near-starvation rate (32). The trade-off between resource use efficiency and growth rate explains the dominance of bacteria harboring a low *rrn* operon copy number as the result of a negative selection for fast-growing bacteria (43) and the adaptation of bacteria with higher growth efficiency in low-resource environments (42, 44). In line with this, our results showed that bacteria with a low copy number of *rrn* operons dominated the activated sludge community during periods of optimal performance. Similar results were obtained by Vuono and colleagues, who manipulated the SRT of a full-scale WWTP and showed that phyla abundant at high and moderate SRT, such as *Planctomycetes*, *Chloroflexi*, *Acidobacteria*, and *Nitrospira*, were outcompeted by fast-growing microorganisms that are adapted for high resource utilization, such as *Proteobacteria* upon a shift to very-low-SRT operation (22). A positive linear correlation between resource availability and the average rRNA operon copy number of microbial populations was also observed in anaerobic digesters (45). Comparable trade-offs between bacterial ecological strategies based on *rrn* copy number distribution were uncovered in microbial succession in several habitats (46), including soil microcosms (47) and stream biofilms (48). In all these previous studies, the observations were rationalized using the r-K selection model, based on life history strategies. However, growth traits of an organism cannot be directly inferred from taxon-based classifications, especially when oligotrophic microorganisms may be r-strategists, whereas copiotrophic microorganisms may be K-strategists (14). As a case in point, we found in this study members of the same family that contain different *rrn* copy numbers and are abundant during periods of contrasting stability (see, e.g., *Polyangiaceae* in Fig. S5), confirming the heterogeneity in life history strategies among phylogenetically related taxa (49). The fact that life history traits of microorganisms present throughout disturbed and stable periods were estimated directly using the experimental data is a strength of this paper.

Based on the generalized relationship between *rrn* copy number and bacterial growth rate (42, 50), we anticipated that differences in *rrn* copy number would mirror differences in bacterial growth rate between the disturbed and stable periods. Evaluation of the bacterial growth rates of specific lineages could be performed using a mass balance, if the numbers of bacteria entering and leaving the system are known (17). Alternatively, growth rates can be probed in metagenomic samples from the differences in read coverage across bacterial genomes (51). Indeed, the algorithm iRep is already an established tool for calculating the inferred *in situ* replication rates of bacteria in microbial communities (38, 52–54).

We found in this study that, even though bacteria with higher *rrn* copy number dominated the disturbed period, estimated replication index values were distributed uniformly throughout time, and no differences were observed between disturbed and stable periods nor had any apparent relationship to SRT. We note that even though the large number of data points gives us high confidence in the reliability of our data, we cannot prove that MAGs used for iRep calculation were a truly unbiased representation of the community. Therefore, actual unseen differences in growth rates cannot be entirely ruled out.

Yet bacterial growth traits that contribute to fitness include not only the rate of exponential growth, but also the length of the lag phase (55). It has been shown that the length of the growth lag phase declines with the number of ribosomal operons (43, 50, 56) and that the competitive fitness difference between Escherichia coli strains with different numbers of *rrn* operons depends on the dynamics of nutrient availability, being most pronounced during the lag phase (57). Similarly, computational models and benchtop experiments of pairwise competition between E. coli mutants of the essential enzyme adenylate kinase (Adk) demonstrated that strains with shorter lag time exhibit higher competitive advantage, especially under nutrient-limiting conditions (58). Based on these previous findings, we infer from the results of this study that bacteria with a high *rrn* copy number might obtain a competitive advantage to rapidly adjust to unsteady operating conditions by growing with a shorter lag phase.

We expect that this hypothesis could be confirmed in future research. Direct measurement of the lag phase, using methods such as total viable count, optical density measurements, or even quantitative PCR (qPCR) with primers targeting specific bacterial populations, is not readily attainable in the context of complex microbial communities such as activated sludge. On the other hand, accurate predictions of the lag phase using computational approaches are very difficult to obtain even for simple model systems (59).

One of the objectives of shotgun analysis is to uncover possible functional genomic signatures that advance our understanding of the mechanisms that allow bacteria to adapt to contrasting regimes of stability and disturbance in activated sludge and how they were reflected in their genomes. The molecular bases of bacterial response to the environment are still poorly understood. Existing studies are based on the exploration of genomes of marine microorganisms (60, 61). In general terms, we note that functional capabilities did not predict which bacterial populations will dominate periods of disturbance or stability. On the contrary, MAGs belonging to each of the contrasting operational periods were not necessarily functionally related. Analysis of MAGs belonging to disturbed and stable periods, based on the presence or absence of metabolic pathways inferred from the KEGG database, indicated that MAGs clustered by phylogeny, rather than by the operational regime to which they belong (Fig. S5). The failure to identify a prototypical genome content that allows clear distinctions between copiotrophic or oligotrophic bacteria was discussed previously by Roller et al. (42). Yet we observed differences at the genomic level that are consistent with recently proposed models, which predict that competitive advantage of bacteria under changing environmental condition is achieved by reserve capacity of ribosomes and transporters. Accordingly, subsaturation allows bacteria to rapidly adapt in fluctuating environments by producing new ribosomes and high-affinity transporters for optimal growth (62, 63). We also observed that the disturbed period was significantly enriched in secretion systems, which are used to deliver a variety of different proteins, including bacterial toxins and degradative enzymes such as proteases and lipases (64). Two-component systems, used by bacteria to detect changes in their environment (65), were also overrepresented in the disturbed period. On the other hand, the stable period contained a significantly higher proportion of genes coding for increased production of secondary metabolites, which are characteristics of organisms with a slow but efficient lifestyle (60).

It may be argued that the nature and frequency of the disturbances occurring in our field-scale experiment were relatively ill-defined. In general, disturbances can be difficult to define, especially for observational studies (8, 66). Here, we concur with the definition of disturbance recently put forward by Cante for use in microbial ecology: “A discrete, unpredictable event that causes direct removal of living biomass, thereby altering community structure” (67). Of interest is that this definition refers to discrete and unpredictable events and that it does not include the notion of disturbance as a rare or relatively infrequent event. In our experiment, disturbance was a random factor that encompassed several events, although the length of the interval between disturbances exceeded the short generation times of microorganisms.

In summary, our results show that disturbances, defined by short-term interruption of wastewater feed supply, fluctuations in MLSS concentration, and/or eventual periods of very low dissolved oxygen concentration, increase the relative proportion of bacteria with higher rRNA operon copy number. Given that the *rrn* operon copy number is considered to reflect ecological strategies in bacteria by influencing their growth traits, we suggest that the length of the growth lag is of primary importance for the capacity of bacteria to thrive under disturbance. We propose that the system has the capacity to maintain its function in the face of disturbance (ecological resilience) through the selection of bacteria that able to return rapidly to their equilibrium or steady-state condition (engineering resilience). Future experiments examining the time course of microbial composition after disturbance using shorter sampling intervals could provide a direct experimental test for the hypothesis put forward in this study: i.e., that a shorter lag phase provided bacteria an advantageous trait in a disturbed environment.

## MATERIALS AND METHODS

### Sample collection.

Samples were obtained from a full-scale municipal WWTP, located in the metropolitan area of Buenos Aires (Argentina), which provides preliminary, primary and secondary treatment to remove organic matter and suspended solids from sewage for a population of 600,000 residents. The WWTP has a modular design, with a capacity of each module to treat 78,000 m^3^/day. Primary effluent receives biological secondary treatment by an activated sludge process in four aeration basins and four secondary clarifiers. At the time sampling started, only one module was in operation. Work on the WWTP upgrade started shortly after the beginning of the sampling period. The transition period was marked by events of operational anomalies, especially caused by temporary shutdown (for several hours) of one or more processes of the WWTP, necessary for civil engineering works. Start-up of the second module was carried out gradually, according to the increase in total influent flow. Throughout the entire sampling period, the plant achieved satisfactory biochemical oxygen demand (BOD) removal, producing an effluent complying with the local effluent-quality regulation.

A total of 60 samples of activated sludge were collected biweekly over a period of 3 years (beginning on November 2012) from one of the aeration basins. Samples were transported within 2 h from the sewage plant to the laboratory and stored at −70°C until DNA extraction.

### DNA extraction and sequencing.

Total DNA from sludge samples was isolated by a direct lysis procedure involving physical disruption of cells and a CTAB (cetyltrimethylammonium bromide) method as described in reference 68. The 60 DNA samples extracted from sludge were sent to INDEAR, Rosario, Argentina, for Nextera DNA library preparation and sequencing. A rapid-run sequencing on two lanes was performed in a HiSeq 1500 Illumina, generating paired-end (PE) reads of 250 bp. The same DNA samples were also sent to Macrogen, Inc., South Korea, for 16S rRNA gene amplicon sequencing. Amplicons of the V3-V4 region were sequenced using Illumina MiSeq, generating PE reads of 300 bp, using the primers b341F (5′CCTACGGGNGGCWGCAG-3′) and Bakt805R (5′-GACTACHVGGGTATCTAATCC-3′) (69).

### 16S rRNA amplicon sequencing analysis.

Amplicon sequencing of the activated sludge samples resulted in 7,293,800 PE reads. After sequence quality controls, raw reads were filtered with Trimmomatic (70) using recommended quality parameters for PE reads and removing reads below 200 bases. A total of 4,581,297 PE reads passed the quality filters. USEARCH v8.1.1861 (71) was used to join the paired sequences, considering a minimum overlap of 32 bp. The USEARCH pipeline was also used to filter merged sequences and define operational taxonomic units (OTUs) at 97% similarity. For diversity analysis, samples were rarified to the lowest number of sequences (11,810). Sequences were classified with Silva database v.132 (72), using 88% minimum identity with the query sequence.

### Metagenome assembly and binning.

Metagenomic sequencing of the activated sludge samples resulted in 328 million PE reads (an average of 5.5 million per sample). PE reads (2 × 250 bp) were filtered and trimmed to remove ambiguous bases (N) and ensured a minimum average quality (*q*) value of 30. After quality filtering, approximately 35% of reads were removed, and the remaining reads were used for the assembly. The filtered reads of the 60 samples were combined into a single file and assembled using MEGAHIT (73), with multiples k-mer length (95 to 99) and default parameters, generating 409,381 contigs longer than 1,500 bp. In order to assemble individual genomes from the contig pool, contigs were binned with MetaBAT (74), using tetranucleotide frequency and coverage information in each of the 60 samples. Binning was manually refined using Cytoscape (75) to visualize the following three criteria: (i) GC percentages, (ii) tetranucleotide frequency distribution, and (iii) abundance profiles along the 60 samples. Manual binning of scaffolds consisted of clustering scaffolds (i.e., considered a bin) when the composite sequences contained ±5% of average percentage of GC, and all scaffolds were interconnected according to Pearson's significant positive correlations (based on tetranucleotide frequency distribution and abundance profiles). As a final step, the reads mapping the contigs from each bin were extracted and reassembled using MEGAHIT as described above. In each sample, approximately one-half of the reads that assembled in contigs (>1,000 bp) were mapped to metagenome-assembled genomes (median, 48.9%; maximum, 59.7%; minimum, 31.2%) (see Fig. S7 in the supplemental material). CheckM (76) was used to estimate the quality (completeness and contamination) of the metagenome-assembled genomes (MAGs).

10.1128/mSystems.00169-19.7 FIG S7 Percentage of reads in each sample that mapped to metagenomic assembled contigs longer than 1,000 bp. Red bars represent the percentage of reads that mapped to MAGs (>60% completeness and <5% contamination). Download FIG S7, EPS file, 0.04 MB. Copyright © 2019 Pérez et al. 2019 Pérez et al. This content is distributed under the terms of the Creative Commons Attribution 4.0 International license.

To estimate the abundance per sample, filtered PE reads were mapped back to the contigs using Bowtie2 (77). The coverage was calculated using MetaBAT (jgi_summarize_bam_contig_depths script). Taxonomic assignment of bacterial genomes was done using the software toolkit GTDB-Tk v.0.1.3 (78) and default parameters.

### Number of rRNA operons (*rrn*).

We applied an approach for quantifying *rrn* copy numbers in MAGs that was independent of the presence of *rrn* operons in the assembled genomes. Reads corresponding to the 16S rRNA gene were identified from raw data from the 60 metagenomic samples using Metaxa2 (79) and assembled *de novo* using EMIRGE (40). Metagenome-assembled 16S rRNA genes (minimum of 900 nucleotides [nt]; called “MA16S” by analogy to MAG) were classified using the Silva database v.132 (72), using 88% minimum identity with the query sequence. Filtered PE reads were mapped back to the MA16S with Bowtie2, and the coverage per sample was estimated using MetaBAT as described above. We applied several filtering criteria to ensure the most accurate matching between MA16S and MAGs for use in downstream analyses. MAGs were initially matched to their corresponding 16S rRNA by Pearson correlation (*r* > 0.7, *P* < 0.001) between the coverage of the MAG and the coverage of MA16S along the entire time series. Second, taxonomic classifications based on the 16S rRNA sequence and on the genome phylogeny had to be concurrent at the highest possible taxonomic resolution. Further support was obtained by sequence match between assembled 16S rRNA genes and contigs in the MAG using BLAST (98% similarity; alignment length of >100 nt; mismatches, <5). Additionally, we searched for the presence of at least five paired-end reads connecting the 16S rRNA gene sequences assembled from the metagenomes (MA16S) with one or more contigs in the MAG. For all the pairs of MAGs and MA16S that satisfied these criteria, the copy number of rRNA operons per MAG was then inferred from the ratio of the coverage of the MA16S to the coverage of the MAG.

To verify that the results obtained from the assembled genomes were not biased by the use of a limited set of MAGs, the *rrn* copy number was also calculated using the whole-metagenome data sets, as follows. The total coverage of genomes in each data set was estimated from the coverage of single-copy universal marker genes, identified in contigs of >500 bp by utilizing hidden Markov models (80). Therefore, the average copy number of rRNA operons (*rrn*) per genome was calculated as the ratio of the coverage of total rRNA SSU genes (MA16S) to the total coverage of a marker gene. This calculation was repeated for a set of 13 single-copy universal marker genes shorter than 450 bp (150 amino acids [aa]).

### Local similarity analysis.

Local similarity analyses (LSA) (81) among the most abundant OTUs (relative abundance average of >0.05%) was used to determine associations and co-occurrence between bacterial species. Local correlations with a score lower than −0.6 and higher than 0.6 and a *q* value of <0.01 were considered significant. The same procedure was applied to determine associations and co-occurrence between MAGs. Gephi software (82) was used to visualize and model the networks.

### Self-organizing maps.

Self-organizing maps (SOMs) were constructed using the R-package SOMbrero (83). SOM analyses were used to cluster the 60 activated sludge samples using two different criteria: (i) abundance patterns of KEGG pathway modules (84) in the whole metagenome and (ii) MAG abundance. For the analysis of functional modules, genes in contigs longer than 2 kbp were predicted using the GeneMark software (85) and annotated with the GhostKOALA tool (86). Only prokaryotic modules identified as complete by the KEGG Mapper—Reconstruct Module tool (87) were used. The abundance of the pathway modules was calculated for each sample as the sum of the abundances of the genes that composed that module.

To identify the KEGG modules and MAGs showing significant differences (*P* < 0.05) between SOM clusters, two group comparisons (*t* test) were performed using the STAMP software (88).

### *In situ* growth rate determination.

*In situ* growth rate of bacteria in the activated sludge system was calculated using the replication index (iRep), a recently developed method that allows the direct estimation of bacterial replication rates from draft-quality genomes assembled from metagenome sequences. The index is estimated from the difference of coverage between the regions of the genome close to the single replication origin and those of the rest of the genome (38, 39). We applied the iRep algorithm, using default parameters, for MAGs with less than 175 scaffolds per Mbp that were more than 75% complete, had less than 5% contamination, and had a genome coverage higher than 5 in any individual sample.

### Data availability.

Sequencing data are available at NCBI BioProject under accession no. PRJNA484416.
