# Supplementary material for: Time Series Genome-Centric Analysis Unveils Bacterial Response to Operational Disturbance in Activated Sludge
Source: mSystems. 2019 Jul 2;4(4):e00169-19. doi: 10.1128/mSystems.00169-19 (PMC6606829; doi:10.1128/mSystems.00169-19)
Supplement: TABLE S1 [file mSystems.00169-19-st001.docx]

Table S1: Operational parameters and treatment performance of activated sludge wastewater treatment plant.

| **Parameter** | **Unit** | **Mean** | **SD** | **Min** | **Max** |
| --- | --- | --- | --- | --- | --- |
| Solids retention time (SRT)^a^ | d | 7.1 | 2.0 | 3.8 | 8.5 |
| Hydraulic retention time | h | 3.4 | 0.9 | 2.2 | 6.0 |
| Influent flow rate | m^3^/d | 65 369 | 28 180 | 31 056 | 134 556 |
| Temperature | °C | 22.0 | 2.9 | 13.2 | 28.7 |
| pH |  | 7.3 | 0.1 | 7.0 | 7.8 |
| Mixed liquor suspended solids ^a^ | mg/l | 1739 | 526 | 360 | 2420 |
| COD influent | mg/l | 384 | 121 | 129 | 676 |
| COD effluent | mg/l | 50 | 13 | 8 | 113 |
| BOD_5_ influent | mg/l | 176 | 59 | 51 | 322 |
| BOD_5_ effluent | mg/l | 17 | 11 | <10 | 48.0 |
| TKN influent ^b^ | mg/l | 25.0 | 6.9 | 6.1 | 45.0 |
| TKN effluent ^b^ | mg/l | 11.2 | 8.1 | 3.0 | 29.0 |
| NH_4_^+^ influent ^b^ | mg/l | 16.5 | 6.4 | 3.3 | 30.0 |
| NH_4_^+^ effluent ^b^ | mg/l | 7.9 | 5.8 | 3.0 | 23.0 |
| NO_3_^-^ influent ^b^ | mg/l | 1.74 | 1.77 | 0.50 | 9.60 |
| NO_3_^-^ effluent ^b^ | mg/l | 7.4 | 4.6 | 0.9 | 20.1 |
| NO_2_^-^ influent ^b^ | mg/l | 0.043 | 0.061 | 0.004 | 0.310 |
| NO_2_^-^ effluent ^b^ | mg/l | 1.34 | 2.07 | 0.02 | 8.70 |
| P influent ^b^ | mg/l | 3.24 | 1.06 | 0.56 | 5.00 |
| P effluent ^b^ | mg/l | 2.01 | 1.60 | 0.63 | 10.00 |
| ^a^ Calculated using data based on a 28-day moving average  ^b^  Values were averaged from monthly data | | | | | |
