# Supplementary material for: Time Series Genome-Centric Analysis Unveils Bacterial Response to Operational Disturbance in Activated Sludge
Source: mSystems. 2019 Jul 2;4(4):e00169-19. doi: 10.1128/mSystems.00169-19 (PMC6606829; doi:10.1128/mSystems.00169-19)
Supplement: TABLE S2 [file mSystems.00169-19-st002.docx]

Table S2: Metagenome-assembled genomes (MAGs) information.

| **MAG**  **ID** | **Length**  **(bp)** | **N°**  **contigs** | **GC (%)** | **Compl**  **(%)** | **Contam**  **(%)** | **Taxonomic classification** |
| --- | --- | --- | --- | --- | --- | --- |
| 1_Bin125 | 3427084 | 50 | 37.58 | 100 | 0 | Bacteroidota; Bacteroidia; AKYH767-A; OLB10 |
| 1_Bin71 | 4282435 | 53 | 41.02 | 99.73 | 0 | Bacteroidota; Bacteroidia; Cytophagales; Cyclobacteriaceae; ELB16-189 |
| 1_Bin69 | 4329948 | 110 | 45.53 | 99.51 | 0.49 | Bacteroidota; Bacteroidia; Chitinophagales; UBA2359 |
| 1_Bin40 | 4293708 | 174 | 67.35 | 99.3 | 3.74 | Proteobacteria; Alphaproteobacteria; Rhizobiales; Beijerinckiaceae; |
| 3_Bin132 | 3346140 | 43 | 65.56 | 99.21 | 0.22 | Proteobacteria; Alphaproteobacteria; Sphingomonadales; Sphingomonadaceae; Novosphingobium |
| 1_Bin78 | 4108406 | 46 | 63.47 | 99.1 | 0.85 | Proteobacteria; Alphaproteobacteria; Rhizobiales; Xanthobacteraceae; P52-10 |
| 1_Bin101 | 3758348 | 153 | 59.95 | 99.05 | 0 | Proteobacteria; Gammaproteobacteria; Betaproteobacteriales; Rhodocyclaceae |
| 1_Bin163 | 2989035 | 19 | 64.84 | 99.01 | 0.73 | Proteobacteria; Alphaproteobacteria; Sphingomonadales; Sphingomonadaceae; Novosphingobium |
| 1_Bin82 | 4155102 | 170 | 39.02 | 99.01 | 1.48 | Bacteroidota; Bacteroidia; Chitinophagales; UBA2359 |
| 1_Bin16 | 7230235 | 138 | 51.94 | 98.75 | 1.18 | Planctomycetota; Planctomycetes; Pirellulales; Pirellulaceae; Pirellula |
| 1_Bin93 | 3974298 | 79 | 44.22 | 98.64 | 0.17 | Proteobacteria; Gammaproteobacteria; Thiotrichales; Thiotrichaceae; Thiothrix_A |
| 1_Bin41 | 5137547 | 62 | 66.42 | 98.58 | 0.66 | Actinobacteriota; Actinobacteria; Corynebacteriales; Corynebacteriaceae; Gordonia |
| 1_Bin147 | 3161810 | 37 | 36.11 | 98.57 | 0.48 | Bacteroidota; Bacteroidia; AKYH767-A; OLB10; OLB10 |
| 3_Bin28 | 5806180 | 202 | 70.66 | 98.54 | 1.06 | Proteobacteria; Alphaproteobacteria; Acetobacterales; Acetobacteraceae; Ga0074136 |
| 1_Bin91 | 3979202 | 107 | 66.05 | 98.39 | 0.25 | Proteobacteria; Alphaproteobacteria; Rhodobacterales; Rhodobacteraceae; UBA1943 |
| 1_Bin70 | 4297487 | 52 | 37.31 | 98.02 | 0.5 | Bacteroidota; Bacteroidia; Chitinophagales; Saprospiraceae; UBA3362 |
| 2_Bin25 | 6074101 | 215 | 36.38 | 97.93 | 1.4 | Bacteroidota; Bacteroidia; Chitinophagales; Saprospiraceae; OLB9 |
| 1_Bin176 | 2824733 | 49 | 32.61 | 97.88 | 0 | Bacteroidota; Bacteroidia; Flavobacteriales; Flavobacteriaceae; Flavobacterium |
| 2_Bin86 | 4085495 | 117 | 66.65 | 97.78 | 0.47 | Proteobacteria; Gammaproteobacteria; Betaproteobacteriales; Burkholderiaceae; Brachymonas |
| 3_Bin64 | 4340068 | 143 | 68.78 | 97.77 | 0.47 | Proteobacteria; Gammaproteobacteria; Betaproteobacteriales; Burkholderiaceae; Rubrivivax |
| 1_Bin150 | 3217468 | 204 | 70.64 | 97.75 | 0.78 | Actinobacteriota; Actinobacteria; Corynebacteriales; Corynebacteriaceae; Dietzia |
| 2_Bin139 | 3250691 | 119 | 30.93 | 97.62 | 0.48 | Bacteroidota; Bacteroidia; AKYH767; b-17BO |
| 1_Bin59 | 3860448 | 89 | 63.99 | 97.48 | 0.8 | Proteobacteria; Alphaproteobacteria; Sphingomonadales; Sphingomonadaceae; Novosphingobium |
| 1_Bin32 | 5632013 | 62 | 64.48 | 97.47 | 0 | Proteobacteria; Alphaproteobacteria; Reyranellales; Reyranellaceae; Reyranella |
| 1_Bin23 | 6274754 | 496 | 63.9 | 97.26 | 3.31 | Proteobacteria; Gammaproteobacteria; Xanthomonadales |
| 1_Bin89 | 3988975 | 298 | 61.77 | 96.67 | 2.87 | Bacteroidota; Bacteroidia; Flavobacteriales; PHOS-HE28; PHOS-HE28 |
| 2_Bin100 | 3891167 | 96 | 64.33 | 96.63 | 0.76 | Proteobacteria; Alphaproteobacteria; Rhodobacterales; Rhodobacteraceae; 32-66-9 |
| 1_Bin102 | 3847345 | 144 | 33.14 | 96.55 | 1.64 | Bacteroidota; Bacteroidia; Chitinophagales; Chitinophagaceae; UBA1930 |
| 5_Bin99 | 3879189 | 97 | 65.17 | 96.55 | 0.3 | Proteobacteria; Alphaproteobacteria; Rhodobacterales; Rhodobacteraceae; 32-66-9 |
| 2_Bin73 | 4217761 | 87 | 45.61 | 96.47 | 0.99 | Bacteroidota; Bacteroidia; Cytophagales; Cyclobacteriaceae; UBA2336 |
| 1_Bin173 | 2879005 | 164 | 58.06 | 96.13 | 0.06 | Proteobacteria; Alphaproteobacteria; Sphingomonadales; Sphingomonadaceae; Ga0077559 |
| 1_Bin52 | 4858650 | 148 | 71.47 | 95.73 | 3.42 | Actinobacteriota; Acidimicrobiia; Microtrichales |
| 1_Bin37 | 5418484 | 366 | 41.93 | 95.71 | 0.33 | Bacteroidota; Bacteroidia; Chitinophagales; Saprospiraceae |
| 2_Bin152 | 3067694 | 205 | 68.14 | 95.63 | 0.78 | Actinobacteriota; Actinobacteria; Propionibacteriales; Nocardioidaceae; Nocardioides |
| 1_Bin229 | 1998143 | 254 | 41.19 | 95.22 | 0.65 | Proteobacteria; Gammaproteobacteria; Pseudomonadales; Moraxellaceae; Acinetobacter |
| 1_Bin35 | 5277815 | 117 | 70.52 | 94.87 | 2.14 | Actinobacteriota; Acidimicrobiia; Microtrichales; Ilumatobacteraceae |
| 1_Bin76 | 4194288 | 93 | 65.55 | 94.87 | 0.85 | Actinobacteriota; Acidimicrobiia; Microtrichales; Microtrichaceae; Microthrix |
| 1_Bin7 | 12030210 | 366 | 70.81 | 94.84 | 4.87 | Myxococcota; Polyangia; Polyangiales; Polyangiaceae; Minicystis |
| 2_Bin138 | 3213126 | 464 | 66.39 | 94.65 | 0.25 | Proteobacteria; Gammaproteobacteria; Betaproteobacteriales; Burkholderiaceae; Hydrogenophaga |
| 2_Bin47 | 3724462 | 154 | 58.83 | 94.63 | 2.47 | Nitrospirota; Nitrospiria; Nitrospirales; Nitrospiraceae; Nitrospira_A |
| 1_Bin162 | 2997686 | 253 | 67.57 | 94.13 | 3.38 | Proteobacteria; Gammaproteobacteria; Xanthomonadales; Xanthomonadaceae; Thermomonas |
| 2_Bin183 | 2601647 | 159 | 61.43 | 94.06 | 0.22 | Proteobacteria; Alphaproteobacteria; Sphingomonadales; Sphingomonadaceae; UBA1936 |
| 2_Bin43 | 5029507 | 358 | 68.59 | 93.85 | 2.18 | Proteobacteria; Gammaproteobacteria; Betaproteobacteriales; Burkholderiaceae |
| 2_Bin120 | 3172205 | 178 | 64.59 | 93.77 | 0.32 | Proteobacteria; Alphaproteobacteria; Sphingomonadales; Sphingomonadaceae; Novosphingobium |
| 2_Bin60 | 4582323 | 247 | 65.52 | 93.7 | 0.52 | Proteobacteria; Alphaproteobacteria; Rhizobiales; Beijerinckiaceae; Rhodoblastus |
| 3_Bin6 | 2830358 | 130 | 62.73 | 93.67 | 0.32 | Proteobacteria; Alphaproteobacteria; Rhizobiales; Xanthobacteraceae |
| 1_Bin223 | 2213419 | 332 | 70.43 | 93.65 | 1.42 | Actinobacteriota; Actinobacteria; Actinomycetales; Microbacteriaceae |
| 1_Bin83 | 3951458 | 349 | 63.17 | 93.61 | 3.24 | Proteobacteria; Alphaproteobacteria; Caulobacterales; Hyphomonadaceae; UBA7672 |
| 1_Bin81 | 4184935 | 257 | 65.04 | 93.34 | 1.51 | Proteobacteria; Alphaproteobacteria; Rhodobacterales; Rhodobacteraceae; UBA1943 |
| 1_Bin119 | 3716657 | 425 | 67.06 | 92.5 | 3.42 | Actinobacteriota; Acidimicrobiia; Microtrichales; Microtrichaceae; UBA11034 |
| 1_Bin84 | 4008020 | 433 | 59.14 | 92.17 | 3.64 | Chloroflexota; Anaerolineae; Caldilineales; Caldilineaceae; UBA5069 |
| 1_Bin123 | 2915873 | 431 | 64.82 | 91.88 | 2.04 | Proteobacteria; Alphaproteobacteria; Sphingomonadales; Sphingomonadaceae |
| 1_Bin15 | 4480915 | 525 | 65.53 | 91.88 | 2.66 | Proteobacteria; Gammaproteobacteria; Betaproteobacteriales; Burkholderiaceae; Rhodoferax |
| 4_Bin48 | 2852766 | 201 | 57.92 | 91.82 | 4.29 | Proteobacteria; Alphaproteobacteria; Sphingomonadales; Sphingomonadaceae; Ga0077559 |
| 3_Bin182 | 2759944 | 216 | 42.57 | 91.61 | 0.3 | Proteobacteria; Alphaproteobacteria; Rhodobacterales; Rhodobacteraceae; QY30 |
| 1_Bin118 | 3587330 | 337 | 35.31 | 91.3 | 0.74 | Bacteroidota; Bacteroidia; Chitinophagales; Chitinophagaceae; OLB11 |
| 1_Bin166 | 2845193 | 326 | 61.39 | 91.2 | 0.85 | Actinobacteriota; Acidimicrobiia; Microtrichales; Microtrichaceae; IMCC26207 |
| 1_Bin180 | 2859416 | 205 | 68.07 | 90.9 | 1.71 | Actinobacteriota; Acidimicrobiia; Microtrichales; Microtrichaceae; IMCC26207 |
| 1_Bin53 | 4793486 | 289 | 69.42 | 90.75 | 1.1 | Proteobacteria; Gammaproteobacteria; Betaproteobacteriales; UKL13-2; |
| 1_Bin5 | 12856257 | 1379 | 71.01 | 90.5 | 3.94 | Myxococcota; Polyangia; Polyangiales; Polyangiaceae |
| 1_Bin77 | 4268067 | 124 | 65.43 | 90.47 | 0.93 | Proteobacteria; Gammaproteobacteria; Chromatiales; Chromatiaceae |
| 1_Bin103 | 3763787 | 355 | 67.31 | 90.37 | 1.21 | Proteobacteria; Alphaproteobacteria; Rhodobacterales; Rhodobacteraceae; 32-66-9 |
| 1_Bin165 | 2921135 | 396 | 60.3 | 90.28 | 0.4 | Proteobacteria; Alphaproteobacteria; Rhizobiales; Hyphomicrobiaceae; Hyphomicrobium |
| 1_Bin11 | 7967773 | 492 | 69.43 | 89.76 | 3 | Myxococcota; Polyangia; Polyangiales; Polyangiaceae |
| 1_Bin126 | 3267519 | 362 | 65.57 | 89.42 | 0.66 | Proteobacteria; Alphaproteobacteria; Rhodobacterales; Rhodobacteraceae |
| 2_Bin21 | 6157184 | 515 | 62.6 | 89.39 | 1.82 | Chloroflexota; Anaerolineae; Caldilineales; Caldilineaceae |
| 3_Bin122 | 2821003 | 266 | 63.01 | 89.38 | 1.04 | Proteobacteria; Gammaproteobacteria; Betaproteobacteriales; Rhodocyclaceae; Dechloromonas |
| 1_Bin198 | 2515157 | 223 | 58.89 | 88.89 | 0.57 | Actinobacteriota; Acidimicrobiia; Microtrichales |
| 2_Bin161 | 3066818 | 277 | 66.63 | 88.79 | 0.85 | Actinobacteriota; Acidimicrobiia; Microtrichales |
| 3_Bin107 | 3571722 | 437 | 67.81 | 88.77 | 1.06 | Proteobacteria; Alphaproteobacteria; Rhodobacterales; Rhodobacteraceae; 24-YEA-8 |
| 1_Bin209 | 2311049 | 360 | 62.33 | 88.75 | 1.72 | Actinobacteriota; Thermoleophilia; Solirubrobacterales; 70-9; 67-14 |
| 2_Bin31 | 5703611 | 675 | 58.64 | 88.73 | 2.45 | Planctomycetota; Planctomycetes; Planctomycetales; Planctomycetaceae |
| 2_Bin27 | 5572021 | 926 | 64.13 | 88.65 | 3.76 | Proteobacteria; Gammaproteobacteria; Xanthomonadales |
| 1_Bin105 | 3735276 | 407 | 60.73 | 88.17 | 1.23 | Bacteroidota; Bacteroidia; Flavobacteriales; PHOS-HE28; PHOS-HE28 |
| 1_Bin110 | 3801240 | 321 | 64.56 | 88.17 | 2.69 | Bacteroidota; Bacteroidia; Flavobacteriales; PHOS-HE28; PHOS-HE28 |
| 1_Bin90 | 3985395 | 467 | 44.57 | 88.14 | 1.23 | Bacteroidota; Bacteroidia; Chitinophagales; Chitinophagaceae; JJ008 |
| 1_Bin80 | 4078682 | 537 | 67.54 | 87.62 | 0.88 | Actinobacteriota; Actinobacteria; Corynebacteriales; Corynebacteriaceae; Mycobacterium |
| 1_Bin104 | 3639612 | 583 | 37.87 | 87.03 | 0.74 | Bacteroidota; Bacteroidia; Chitinophagales; Chitinophagaceae; OLB11 |
| 2_Bin160 | 3054732 | 383 | 66.8 | 86.64 | 2.74 | Proteobacteria; Alphaproteobacteria; Rhizobiales; Beijerinckiaceae |
| 2_Bin94 | 3686314 | 811 | 64.73 | 86.63 | 2.26 | Actinobacteriota; Actinobacteria; Corynebacteriales; Corynebacteriaceae; Mycobacterium |
| 1_Bin197 | 2558251 | 182 | 68.52 | 86.51 | 4.27 | Actinobacteriota; Acidimicrobiia; Microtrichales; Microtrichaceae; IMCC26207 |
| 2_Bin133 | 3339294 | 405 | 65.53 | 86.35 | 2.33 | Proteobacteria; Gammaproteobacteria; Xanthomonadales; UBA4656 |
| 1_Bin61 | 4236871 | 603 | 69.31 | 86.32 | 2.56 | Actinobacteriota; Acidimicrobiia; Microtrichales; Ilumatobacteraceae; UBA8979 |
| 2_Bin143 | 3313055 | 289 | 56.37 | 85.99 | 1.76 | Acidobacteriota; Blastocatellia; Pyrinomonadales; Pyrinomonadaceae; OLB17 |
| 1_Bin19 | 3106552 | 285 | 66.73 | 85.71 | 1.88 | Proteobacteria; Alphaproteobacteria; Sphingomonadales; Sphingomonadaceae; Novosphingobium |
| 1_Bin134 | 3525267 | 416 | 42.29 | 85.26 | 1.98 | Bacteroidota; Bacteroidia; Chitinophagales; Saprospiraceae |
| 1_Bin62 | 4372890 | 165 | 71.69 | 85.2 | 2.29 | Proteobacteria; Gammaproteobacteria; Steroidobacterales; Steroidobacteraceae; UBA964 |
| 1_Bin169 | 2965799 | 447 | 65.69 | 85.04 | 1.3 | Proteobacteria; Alphaproteobacteria; Sphingomonadales; Sphingomonadaceae; Novosphingobium |
| 2_Bin14 | 7484387 | 581 | 42 | 83.65 | 3.1 | Bacteroidota; Bacteroidia; Cytophagales; Spirosomaceae; Runella |
| 1_Bin58 | 4146732 | 818 | 40.9 | 83.36 | 1.51 | Bacteroidota; Bacteroidia; Chitinophagales; UBA2359 |
| 1_Bin128 | 3182753 | 726 | 36.09 | 83.03 | 0.79 | Bacteroidota; Bacteroidia; AKYH767; UBA4408; UBA4408 |
| 1_Bin18 | 6048558 | 400 | 43.4 | 82.97 | 0.6 | Bacteroidota; Bacteroidia; Cytophagales; Spirosomaceae; Runella |
| 1_Bin244 | 2130113 | 263 | 61.84 | 82.86 | 2.68 | Actinobacteriota; Actinobacteria; Propionibacteriales; Nocardioidaceae |
| 1_Bin181 | 2941727 | 410 | 61.64 | 82.21 | 1.97 | Bacteroidota; Bacteroidia; Flavobacteriales; PHOS-HE28; PHOS-HE28 |
| 1_Bin10 | 7905179 | 1574 | 74.02 | 81.65 | 1.29 | Myxococcota; Polyangia; Kofleriales; Kofleriaceae; UBA2376 |
| 3_Bin144 | 3196582 | 494 | 68.52 | 81.45 | 3.05 | Actinobacteriota; Actinobacteria; Actinomycetales; Dermatophilaceae; GCA-2748155 |
| 1_Bin171 | 3101100 | 458 | 37.26 | 81.2 | 1.54 | Bacteroidota; Bacteroidia; Chitinophagales; Saprospiraceae; OLB8 |
| 1_Bin65 | 4415542 | 710 | 70.73 | 81.13 | 3.47 | Acidobacteriota; Luteitaleia; Luteitaleales; UBA2999 |
| 1_Bin172 | 2724079 | 635 | 66.34 | 80.96 | 1.48 | Proteobacteria; Gammaproteobacteria; Xanthomonadales; Rhodanobacteraceae; Dokdonella |
| 2_Bin24 | 6104963 | 980 | 64.48 | 80.96 | 2.73 | Chloroflexota; Anaerolineae; Caldilineales; Caldilineaceae |
| 2_Bin92 | 3396841 | 668 | 62.77 | 80.91 | 1.67 | Proteobacteria; Alphaproteobacteria; Rhodobacterales; Rhodobacteraceae; QY30 |
| 1_Bin121 | 2990410 | 376 | 62.02 | 80.82 | 0.3 | Proteobacteria; Alphaproteobacteria; Rhodobacterales; Rhodobacteraceae; UBA6273 |
| 2_Bin44 | 4732194 | 569 | 74.13 | 80.64 | 1.23 | Proteobacteria; Gammaproteobacteria; Betaproteobacteriales; Burkholderiaceae; UBA4615 |
| 1_Bin12 | 6709626 | 660 | 54.48 | 80.55 | 0 | Planctomycetota; Planctomycetes; Planctomycetales; Planctomycetaceae; UBA10327 |
| 1_Bin51 | 4496455 | 899 | 69.65 | 79.8 | 1.36 | Proteobacteria; Gammaproteobacteria; Betaproteobacteriales; Burkholderiaceae; Leptothrix |
| 1_Bin257 | 1441469 | 326 | 35.07 | 79.61 | 1.64 | Bacteroidota; Bacteroidia; Chitinophagales; Chitinophagaceae |
| 2_Bin145 | 3198467 | 530 | 70.82 | 79.46 | 1.94 | Actinobacteriota; Acidimicrobiia; Microtrichales; Microtrichaceae; IMCC26207 |
| 1_Bin29 | 5770548 | 1180 | 46.62 | 78.95 | 1.77 | Bacteroidota; Bacteroidia; Chitinophagales; Saprospiraceae; Haliscomenobacter |
| 1_Bin129 | 3294665 | 743 | 70.98 | 78.27 | 2.04 | Proteobacteria; Gammaproteobacteria; Betaproteobacteriales; UKL13-2; GR16-43 |
| 1_Bin347 | 790005 | 126 | 50.01 | 77.09 | 2.31 | Patescibacteria; Saccharimonadia; Saccharimonadales; Saccharimonadaceae; Saccharimonas |
| 1_Bin45 | 4999488 | 902 | 69.27 | 76.95 | 2.83 | Acidobacteriota; Luteitaleia; Luteitaleales; Luteitaleaceae |
| 1_Bin205 | 2460759 | 432 | 36.44 | 76.81 | 3.45 | Bacteroidota; Bacteroidia; Chitinophagales; Chitinophagaceae; Sediminibacterium |
| 1_Bin201 | 2774623 | 548 | 66.77 | 76.77 | 2.14 | Proteobacteria; Alphaproteobacteria; Rhizobiales; Beijerinckiaceae |
| 1_Bin98 | 4047705 | 702 | 49.12 | 76.73 | 0.25 | Bacteroidota; Bacteroidia; Chitinophagales; Saprospiraceae; UBA10441 |
| 2_Bin113 | 3302801 | 618 | 38.83 | 75.74 | 0 | Bacteroidota; Bacteroidia; AKYH767-A; OLB10 |
| 3_Bin154 | 3046204 | 678 | 68.45 | 75.01 | 0.91 | Proteobacteria; Alphaproteobacteria; Rhodobacterales; Rhodobacteraceae; 24-YEA-8 |
| 1_Bin350 | 865983 | 88 | 51.26 | 74.55 | 0 | Patescibacteria; Saccharimonadia; UBA4664 |
| 1_Bin112 | 3734690 | 708 | 64.51 | 74.36 | 0.4 | Proteobacteria; Alphaproteobacteria; Rhizobiales; Hyphomicrobiaceae |
| 3_Bin50 | 4936925 | 921 | 49.61 | 74.34 | 0.62 | Bacteroidota; Bacteroidia; Chitinophagales; Saprospiraceae; UBA10441 |
| 2_Bin179 | 3139771 | 485 | 69.2 | 74.2 | 0.72 | Actinobacteriota; Actinobacteria; Actinomycetales; Dermatophilaceae; GCA-2748155 |
| 2_Bin190 | 2343333 | 613 | 48.7 | 73.99 | 1.8 | Bacteroidota; Bacteroidia; Flavobacteriales; Flavobacteriaceae; Flavobacterium |
| 1_Bin135 | 3229177 | 712 | 36.5 | 73.75 | 0.62 | Bacteroidota; Bacteroidia; Chitinophagales; BACL12; UBA7236 |
| 3_Bin48 | 1957969 | 486 | 61.29 | 73.63 | 1.37 | Proteobacteria; Alphaproteobacteria; Sphingomonadales; Sphingomonadaceae; Ga0077559 |
| 3_Bin236 | 2239751 | 392 | 70.84 | 72.99 | 0 | Actinobacteriota; Actinobacteria; Actinomycetales; Dermatophilaceae; Tetrasphaera |
| 1_Bin170 | 2508746 | 615 | 57.88 | 72.87 | 0.94 | Proteobacteria; Alphaproteobacteria; Rhizobiales; Beijerinckiaceae; |
| 1_Bin224 | 2199296 | 511 | 42.02 | 71.98 | 2.96 | Bacteroidota; Bacteroidia; Chitinophagales |
| 2_Bin151 | 3026885 | 335 | 69.42 | 71.6 | 0.5 | Proteobacteria; Gammaproteobacteria; Betaproteobacteriales; Burkholderiaceae |
| 1_Bin33 | 5786162 | 822 | 51.5 | 71.42 | 1.18 | Planctomycetota; Planctomycetes; Pirellulales; Pirellulaceae; UBA10444 |
| 2_Bin203 | 2266746 | 610 | 43.61 | 71.35 | 0.69 | Bacteroidota; Bacteroidia; Chitinophagales; Chitinophagaceae; Ferruginibacter |
| 1_Bin307 | 1086865 | 48 | 45.33 | 71.3 | 0 | Patescibacteria; Saccharimonadia; Saccharimonadales; UBA4665 |
| 1_Bin75 | 3966697 | 668 | 68.99 | 71.18 | 3.08 | Proteobacteria; Gammaproteobacteria; Betaproteobacteriales; Burkholderiaceae; Ideonella_A |
| 1_Bin57 | 4351947 | 1255 | 59.31 | 71.17 | 3.45 | Planctomycetota; Planctomycetes; Planctomycetales; Planctomycetaceae |
| 2_Bin116 | 3667631 | 662 | 69.53 | 70.77 | 4.28 | Proteobacteria; Gammaproteobacteria; Betaproteobacteriales; Burkholderiaceae; Rhodoferax |
| 1_Bin96 | 3818267 | 881 | 39.46 | 70.67 | 1.97 | Bacteroidota; Bacteroidia; Chitinophagales; Chitinophagaceae; UBA3961 |
| 1_Bin68 | 3993087 | 1099 | 64.01 | 70.2 | 0 | Chloroflexota; Anaerolineae |
| 1_Bin199 | 2218410 | 627 | 35.13 | 69.93 | 0.96 | Bacteroidota; Bacteroidia; Flavobacteriales; Flavobacteriaceae; Flavobacterium |
| 1_Bin56 | 4206083 | 1111 | 55.31 | 69.38 | 0 | Planctomycetota; Planctomycetes; Planctomycetales; Planctomycetaceae; UBA10327 |
| 2_Bin131 | 3644790 | 769 | 69.8 | 68.71 | 1.17 | Proteobacteria; Gammaproteobacteria; Betaproteobacteriales; Burkholderiaceae; Rubrivivax |
| 1_Bin271 | 1528399 | 489 | 41.65 | 68.1 | 0.81 | Verrucomicrobiota; Chlamydiia; Parachlamydiales |
| 1_Bin6 | 3154304 | 859 | 63.41 | 67.98 | 2.98 | Proteobacteria; Alphaproteobacteria; Rhizobiales; Beijerinckiaceae; 28-YEA-48 |
| 1_Bin136 | 3293204 | 800 | 53.29 | 67.85 | 1.77 | Bacteroidota; Bacteroidia; Chitinophagales; Saprospiraceae; UBA2329 |
| 1_Bin95 | 3484239 | 1009 | 68.4 | 67.54 | 3.24 | Proteobacteria; Gammaproteobacteria; Betaproteobacteriales; Burkholderiaceae; JOSHI-001 |
| 1_Bin142 | 3137507 | 771 | 72.63 | 67.49 | 2.48 | Actinobacteriota; Acidimicrobiia; Microtrichales |
| 1_Bin346 | 892411 | 16 | 45.89 | 66.82 | 2.78 | Patescibacteria; Saccharimonadia; Saccharimonadales; Saccharimonadaceae |
| 2_Bin87 | 4024885 | 327 | 65.58 | 66.72 | 1.72 | Proteobacteria; Alphaproteobacteria; Rhizobiales; Thermopetrobacteraceae |
| 1_Bin364 | 811432 | 56 | 44.39 | 66.67 | 0.93 | Patescibacteria; Saccharimonadia; Saccharimonadales; UBA4665 |
| 1_Bin9 | 5595356 | 1354 | 63.38 | 65.78 | 1.15 | Planctomycetota; Planctomycetes; Pirellulales; Pirellulaceae; |
| 1_Bin274 | 1676443 | 429 | 45.51 | 65.63 | 0.25 | Bacteroidota; Bacteroidia; Chitinophagales |
| 2_Bin63 | 4902831 | 1076 | 42.1 | 65.44 | 3.87 | Bacteroidota; Bacteroidia; Cytophagales; Spirosomaceae |
| 1_Bin306 | 1135776 | 315 | 46.86 | 65.22 | 1.37 | Firmicutes; Bacilli; Lactobacillales; Aerococcaceae; Trichococcus |
| 1_Bin362 | 801225 | 102 | 37.39 | 64.74 | 0.99 | Patescibacteria; Saccharimonadia; Saccharimonadales |
| 1_Bin189 | 2485192 | 623 | 68.13 | 64.65 | 0.68 | Proteobacteria; Alphaproteobacteria; Sphingomonadales; Sphingomonadaceae; Novosphingobium |
| 1_Bin175 | 3085891 | 440 | 46.25 | 64.45 | 1.02 | Bacteroidota; Bacteroidia; Chitinophagales; Chitinophagaceae; JJ008 |
| 1_Bin355 | 847582 | 27 | 39.51 | 64.42 | 0 | Patescibacteria; Saccharimonadia; Saccharimonadales; UBA7683 |
| 1_Bin208 | 1876468 | 564 | 64.59 | 64.1 | 0.26 | Actinobacteriota; Actinobacteria; Propionibacteriales; Nocardioidaceae |
| 1_Bin336 | 989902 | 17 | 50.53 | 64.04 | 0 | Patescibacteria; Saccharimonadia; Saccharimonadales; Saccharimonadaceae |
| 1_Bin334 | 972267 | 100 | 47.36 | 63.79 | 3.7 | Patescibacteria; Saccharimonadia; Saccharimonadales; Saccharimonadaceae; UBA2112 |
| 1_Bin351 | 802082 | 26 | 47.3 | 63.72 | 0.85 | Patescibacteria; Saccharimonadia; Saccharimonadales; Saccharimonadaceae |
| 1_Bin331 | 1043663 | 60 | 43 | 63.58 | 0 | Patescibacteria; Saccharimonadia; Saccharimonadales |
| 1_Bin141 | 3079045 | 857 | 70.74 | 63.57 | 0.41 | Proteobacteria; Alphaproteobacteria; Acetobacterales; Acetobacteraceae; 70-18 |
| 1_Bin380 | 756713 | 65 | 36.96 | 63.45 | 1.1 | Patescibacteria; Saccharimonadia; Saccharimonadales |
| 2_Bin216 | 2502336 | 661 | 65.83 | 63.15 | 1.27 | Proteobacteria; Alphaproteobacteria; Rhodobacterales; Rhodobacteraceae; UBA1943 |
| 2_Bin219 | 2145308 | 528 | 70.36 | 62.97 | 0.57 | Actinobacteriota; Actinobacteria; Nanopelagicales; S36-B12 |
| 1_Bin361 | 830251 | 70 | 46.62 | 62.75 | 1.85 | Patescibacteria; Saccharimonadia; Saccharimonadales; Saccharimonadaceae; UBA1020 |
| 5_Bin230 | 2418492 | 434 | 66.73 | 62.54 | 0 | Proteobacteria; Gammaproteobacteria; Betaproteobacteriales; Burkholderiaceae |
| 1_Bin370 | 775515 | 143 | 45.64 | 62.24 | 0 | Patescibacteria; Paceibacteria; Moranbacterales; UBA1568; UBA1568 |
| 1_Bin265 | 2016044 | 414 | 64.64 | 61.78 | 0.45 | Proteobacteria; Alphaproteobacteria; Rhodobacterales; Rhodobacteraceae; QY30 |
| 2_Bin215 | 2596077 | 652 | 71.24 | 61.55 | 1.05 | Proteobacteria; Gammaproteobacteria; Betaproteobacteriales; Burkholderiaceae; Sphaerotilus |
| 1_Bin207 | 2251275 | 670 | 53.76 | 61.5 | 2.94 | Spirochaetota; Leptospirae; Turneriellales; Turneriellaceae; Turneriella |
| 1_Bin212 | 2284083 | 623 | 38.94 | 61.2 | 4.68 | Bacteroidota; Bacteroidia; Chitinophagales; LD1; UBA1941 |
| 1_Bin72 | 3857124 | 1097 | 56.62 | 60.81 | 0 | Planctomycetota; Planctomycetes; Planctomycetales; Planctomycetaceae |
| 2_Bin276 | 1904600 | 341 | 69.13 | 60.67 | 0 | Actinobacteriota; Actinobacteria; Actinomycetales; Dermatophilaceae; Tetrasphaera |
| 1_Bin74 | 3530063 | 1137 | 69.62 | 60.55 | 0.85 | Acidobacteriota; Thermoanaerobaculia; UBA5066; UBA5066; UBA5066 |
