# Supplementary material for: Time Series Genome-Centric Analysis Unveils Bacterial Response to Operational Disturbance in Activated Sludge
Source: mSystems. 2019 Jul 2;4(4):e00169-19. doi: 10.1128/mSystems.00169-19 (PMC6606829; doi:10.1128/mSystems.00169-19)
Supplement: TABLE S3 [file mSystems.00169-19-st003.docx]

Table S3: MAGs and MA16S data used for the calculation of ribosomal RNA operon copy numbers.

| **MAGs** | | | **EMIRGE 16S rRNA gene reconstruction (MA16s)** | | | **rrn copy number estimation ^b^** | | |
| --- | --- | --- | --- | --- | --- | --- | --- | --- |
| **ID** | **Taxonomic classification** | **rrnDB^a^ (Mode)** | **ID** | **Length** | **Taxonomic classification (SILVA v132 95% identity)** | **copy number** | **R** | **p** |
| 1_Bin101 | Proteobacteria; Gammaproteobacteria; Betaproteobacteriales; **Rhodocyclaceae**; | 2-4 | 766_EF565157.1.1533 | 1424 | Proteobacteria; Gammaproteobacteria; Betaproteobacteriales; Rhodocyclaceae; Candidatus Accumulibacter | 3 | 0.91 | <0.001 |
| 1_Bin11 | Myxococcota; Polyangia; Polyangiales; **Polyangiaceae** | 3-4 (4) | 869_HQ111161.1.1518 | 1496 | Proteobacteria; Deltaproteobacteria; Myxococcales; Polyangiaceae; Pajaroellobacter | 2 | 0.96 | <0.001 |
| 1_Bin118 | Bacteroidota; Bacteroidia; Chitinophagales; **Chitinophagaceae**; OLB11 | 1-6 | 316_FM201197.1.1391 | 1348 | Bacteroidetes; Bacteroidia; Chitinophagales; Chitinophagaceae | 1 | 0.92 | <0.001 |
| 1_Bin12 | Planctomycetota; Planctomycetes; Planctomycetales; **Planctomycetaceae**; UBA10327 | 1-4 (2) | 584_GQ994737.1.1317 | 1298 | Planctomycetes; Planctomycetacia; Planctomycetales; Rubinisphaeraceae | 2 | 0.86 | <0.001 |
| 1_Bin125 | Bacteroidota; **Bacteroidia**; AKYH767-A; OLB10 | 2-7 (4) | 2208_FJ660501.1.1485 | 1427 | Bacteroidetes; Bacteroidia; Sphingobacteriales; AKYH767 | 1 | 0.99 | <0.001 |
| 1_Bin134 | Bacteroidota; Bacteroidia; Chitinophagales; **Saprospiraceae** | 3 | 105_HQ609699.1.1484 | 1434 | Bacteroidetes; Bacteroidia; Chitinophagales; Saprospiraceae | 3 | 0.93 | <0.001 |
| 1_Bin147 | Bacteroidota; **Bacteroidia**; AKYH767-A; OLB10; OLB10 | 2-7 (4) | 552_JN391906.1.1488 | 1186 | Bacteroidetes; Bacteroidia; Sphingobacteriales; AKYH767 | 1 | 0.94 | <0.001 |
| 1_Bin150 | Actinobacteriota; Actinobacteria; Corynebacteriales; Corynebacteriaceae; **Dietzia** | 2-4 | 154_AF513094.1.1447 | 1225 | Actinobacteria; Actinobacteria; Corynebacteriales; Dietziaceae; Dietzia | 2 | 0.79 | <0.001 |
| 1_Bin16 | Planctomycetota; Planctomycetes; Pirellulales; Pirellulaceae; **Pirellula** | 1-2 (1) | 926_JN391698.1.1487 | 1316 | Planctomycetes; Planctomycetacia; Pirellulales; Pirellulaceae; Pirellula | 1 | 0.96 | <0.001 |
| 1_Bin171 | Bacteroidota; Bacteroidia; Chitinophagales; **Saprospiraceae**; OLB8 | 3 | 24_GU454872.1.1490 | 1465 | Bacteroidetes; Bacteroidia; Chitinophagales; Saprospiraceae; OLB8 | 3 | 0.96 | <0.001 |
| 1_Bin175 | Bacteroidota; Bacteroidia; Chitinophagales; **Chitinophagaceae**; JJ008 | 1-6 | 1664_AB286400.1.1455 | 1394 | Bacteroidetes; Bacteroidia; Chitinophagales; Chitinophagaceae; Terrimonas | 1 | 0.86 | <0.001 |
| 1_Bin176 | Bacteroidota; Bacteroidia; Flavobacteriales; Flavobacteriaceae; **Flavobacterium** | 1-13 (6) | 976_JN391970.1.1473 | 1235 | Bacteroidetes; Bacteroidia; Flavobacteriales; Flavobacteriaceae; Flavobacterium | 4 | 0.90 | <0.001 |
| 1_Bin199 | Bacteroidota; Bacteroidia; Flavobacteriales; Flavobacteriaceae; **Flavobacterium** | 1-13 (6) | 811_GU295969.1.1408 | 1185 | Bacteroidetes; Bacteroidia; Flavobacteriales; Flavobacteriaceae; Flavobacterium | 4 | 0.86 | <0.001 |
| 1_Bin205 | Bacteroidota; Bacteroidia; Chitinophagales; **Chitinophagaceae**; Sediminibacterium | 1-6 | 1065_HM128915.1.1461 | 1319 | Bacteroidetes; Bacteroidia; Chitinophagales; Chitinophagaceae; Sediminibacterium | 3 | 0.94 | <0.001 |
| 1_Bin212 | Bacteroidota; Bacteroidia; **Chitinophagales**; LD1; UBA1941 | 1-6 | 358_JN391574.1.1488 | 1225 | Bacteroidetes; Bacteroidia; Chitinophagales; 37-13 | 2 | 0.95 | <0.001 |
| 1_Bin23 | Proteobacteria; Gammaproteobacteria; **Xanthomonadales** | 1-4 (2) | 158_AB478683.1.1505 | 1490 | Proteobacteria; Gammaproteobacteria; Xanthomonadales; Rhodanobacteraceae | 2 | 0.95 | <0.001 |
| 1_Bin306 | Firmicutes; Bacilli; Lactobacillales; **Aerococcaceae**; Trichococcus | 4-7 (4) | 1555_GQ249358.1.1505 | 1476 | Firmicutes; Bacilli; Lactobacillales; Carnobacteriaceae; Trichococcus | 10 | 0.89 | <0.001 |
| 1_Bin32 | Proteobacteria; **Alphaproteobacteria**; Reyranellales; Reyranellaceae; Reyranella | 1-11 (3) | 3120_HE583160.1.1379 | 1053 | Proteobacteria; Alphaproteobacteria; Reyranellales; Reyranellaceae; Reyranella | 1 | 0.94 | <0.001 |
| 1_Bin33 | Planctomycetota; **Planctomycetes**; Pirellulales; Pirellulaceae; UBA10444 | 1-8 (1) | 315_GU455196.1.1482 | 1468 | Planctomycetes; Planctomycetacia; Pirellulales; Pirellulaceae; Pirellula | 1 | 0.79 | <0.001 |
| 1_Bin350 | Patescibacteria; Saccharimonadia; UBA4664 | na | 3447_AB286434.1.1391 | 1193 | Patescibacteria; Saccharimonadia; Saccharimonadales | 1 | 0.97 | <0.001 |
| 1_Bin355 | Patescibacteria; Saccharimonadia; Saccharimonadales; UBA7683 | na | 135_AF268999.1.1429 | 1421 | Patescibacteria; Saccharimonadia; Saccharimonadales | 1 | 0.99 | <0.001 |
| 1_Bin362 | Patescibacteria; Saccharimonadia; Saccharimonadales | na | 1775_JN398100.1.1398 | 1356 | Patescibacteria; Saccharimonadia; Saccharimonadales | 1 | 0.93 | <0.001 |
| 1_Bin380 | Patescibacteria; Saccharimonadia; Saccharimonadales | na | 554_HM773452.1.1439 | 1375 | Patescibacteria; Saccharimonadia; Saccharimonadales | 1 | 0.97 | <0.001 |
| 1_Bin40 | Proteobacteria; Alphaproteobacteria; Rhizobiales; **Beijerinckiaceae** | 2-3 | 34_CU919262.1.1305 | 1266 | Proteobacteria; Alphaproteobacteria; Rhizobiales; Beijerinckiaceae | 3 | 0.99 | <0.001 |
| 1_Bin41 | Actinobacteriota; Actinobacteria; Corynebacteriales; Corynebacteriaceae; **Gordonia** | 2-4 (3) | 21_CU926110.1.1340/  188_EU041713.1.1413 | 1335/  1344 | Actinobacteria; Actinobacteria; Corynebacteriales; Nocardiaceae; Gordonia | 5 | 0.98/  0.97 | <0.001 |
| 1_Bin5 | Myxococcota; Polyangia; Polyangiales; **Polyangiaceae** | 3-4 (4) | 4664_HQ450133.1.1482 | 1165 | Proteobacteria; Deltaproteobacteria; Myxococcales; Polyangiaceae | 1 | 0.98 | <0.001 |
| 1_Bin65 | **Acidobacteriota**; Luteitaleia; Luteitaleales; UBA2999 | 1-2 (1) | 831_AF234731.1.1550 | 1322 | Acidobacteria; Subgroup 6 | 2 | 0.93 | <0.001 |
| 1_Bin69 | Bacteroidota; Bacteroidia; **Chitinophagales**; UBA2359 | 1-6 | 2222_JN609375.1.1478 | 1468 | Bacteroidetes; Bacteroidia; Chitinophagales; 37-13 | 1 | 0.99 | <0.001 |
| 1_Bin7 | Myxococcota; Polyangia; Polyangiales; Polyangiaceae; **Minicystis** | 4 | 783_EU662572.1.1510 | 1496 | Proteobacteria; Deltaproteobacteria; Myxococcales; Polyangiaceae | 2 | 0.96 | <0.001 |
| 1_Bin70 | Bacteroidota; Bacteroidia; Chitinophagales; **Saprospiraceae**; UBA3362 | 3 | 2076_JN391572.1.1497 | 1464 | Bacteroidetes; Bacteroidia; Chitinophagales; Saprospiraceae | 2 | 0.99 | <0.001 |
| 1_Bin71 | Bacteroidota; Bacteroidia; Cytophagales; **Cyclobacteriaceae**; ELB16-189 | 3-4 (4) | 254_JN679190.1.1489 | 1384 | Bacteroidetes; Bacteroidia; Cytophagales; Microscillaceae; OLB12 | 2 | 0.93 | <0.001 |
| 1_Bin76 | Actinobacteriota; Acidimicrobiia; Microtrichales; Microtrichaceae; Microthrix | na | 163_CU924480.1.1336 | 1117 | Actinobacteria; Acidimicrobiia; Microtrichales; Microtrichaceae; Candidatus Microthrix | 1 | 0.99 | <0.001 |
| 1_Bin77 | Proteobacteria; Gammaproteobacteria; Chromatiales; **Chromatiaceae** | 1-3 (2) | 259_HQ609657.1.1500 | 1302 | Proteobacteria; Gammaproteobacteria; Run-SP154 | 2 | 0.98 | <0.001 |
| 1_Bin78 | Proteobacteria; Alphaproteobacteria; Rhizobiales; **Xanthobacteraceae**; P52-10 | 1-3 | 692_GU455342.1.1451/  1357_CU924996.2.1311 | 1412/  1249 | Proteobacteria; Alphaproteobacteria; Rhizobiales; Xanthobacteraceae | 4 | 0.83 | <0.001 |
| 1_Bin82 | Bacteroidota; Bacteroidia; **Chitinophagales**; UBA2359 | 1-6 | 2308_JF828737.1.1489 | 1486 | Bacteroidetes; Bacteroidia; Chitinophagales; 37-13 | 1 | 0.99 | <0.001 |
| 1_Bin93 | Proteobacteria; Gammaproteobacteria; Thiotrichales; **Thiotrichaceae**; Thiothrix_A | 1-2 (2) | 118_AATN01000498.849.2327 | 1471 | Proteobacteria; Gammaproteobacteria; Thiotrichales; Thiotrichaceae; Thiothrix | 1 | 0.99 | <0.001 |
| 1_Bin98 | Bacteroidota; Bacteroidia; Chitinophagales; **Saprospiraceae**; UBA10441 | 3 | 309_EU104080.1.1428 | 1165 | Bacteroidetes; Bacteroidia; Chitinophagales; Saprospiraceae | 2 | 0.97 | <0.001 |
| 2_Bin100 | Proteobacteria; Alphaproteobacteria; Rhodobacterales; **Rhodobacteraceae**; 32-66-9 | 1-6 (4) | 590_CU927633.1.1283 | 1278 | Proteobacteria; Alphaproteobacteria; Rhodobacterales; Rhodobacteraceae; Rhodobacter | 2 | 0.78 | <0.001 |
| 2_Bin133 | Proteobacteria; Gammaproteobacteria; **Xanthomonadales**; UBA4656 | 1-4 (2) | 1896_JN391783.1.1509 | 1148 | Proteobacteria; Gammaproteobacteria; Xanthomonadales; Rhodanobacteraceae | 3 | 0.95 | <0.001 |
| 2_Bin139 | Bacteroidota; **Bacteroidia**; AKYH767; b-17BO | 2-7 (4) | 2279_GQ389162.1.1482 | 1412 | Bacteroidetes; Bacteroidia; Sphingobacteriales; env.OPS 17 | 2 | 0.93 | <0.001 |
| 2_Bin143 | Acidobacteriota; **Blastocatellia**; Pyrinomonadales; Pyrinomonadaceae; OLB17 | 1 | 1063_JN391683.1.1486 | 1367 | Acidobacteria; Blastocatellia (Subgroup 4); Blastocatellales; Blastocatellaceae; JGI 0001001-H03 | 2 | 0.87 | <0.001 |
| 2_Bin161 | Actinobacteriota; Acidimicrobiia; Microtrichales | na | 486_JN609378.1.1484 | 1462 | Actinobacteria; Acidimicrobiia; Microtrichales; Iamiaceae; Iamia | 1 | 0.87 | <0.001 |
| 2_Bin215 | Proteobacteria; Gammaproteobacteria; Betaproteobacteriales; **Burkholderiaceae**; Sphaerotilus | 1-7 (4) | 382_JN391803.1.1495 | 1486 | Proteobacteria; Gammaproteobacteria; Betaproteobacteriales; Burkholderiaceae | 3 | 0.95 | <0.001 |
| 2_Bin216 | Proteobacteria; Alphaproteobacteria; Rhodobacterales; **Rhodobacteraceae**; UBA1943 | 1-6 (4) | 1358_CU920600.1.1317/  1461_CU920200.1.1285 | 900/  1225 | Proteobacteria; Alphaproteobacteria; Rhodobacterales; Rhodobacteraceae; Rhodobacter | 2 | 0.94/  0.94 | <0.001 |
| 2_Bin31 | Planctomycetota; Planctomycetes; Planctomycetales; **Planctomycetaceae**; | 1-4 (2) | 319_JN391700.1.1465 | 1364 | Planctomycetes; Planctomycetacia; Planctomycetales; Rubinisphaeraceae; SH-PL14 | 3 | 0.77 | <0.001 |
| 2_Bin60 | Proteobacteria; Alphaproteobacteria; Rhizobiales; **Beijerinckiaceae**; Rhodoblastus | 2-3 | 51_HQ538633.1.1442 | 1410 | Proteobacteria; Alphaproteobacteria; Rhizobiales; Beijerinckiaceae; Methylocystis | 1 | 0.95 | <0.001 |
| 2_Bin63 | Bacteroidota; Bacteroidia; **Cytophagales**; Spirosomaceae | 1-8 (3) | 176_EU104183.1.1430 | 1414 | Bacteroidetes; Bacteroidia; Cytophagales; Spirosomaceae | 4 | 0.91 | <0.001 |
| 2_Bin73 | Bacteroidota; Bacteroidia; Cytophagales; **Cyclobacteriaceae**; UBA2336 | 3-4 (4) | 416_HQ827948.1.1448 | 1429 | Bacteroidetes; Bacteroidia; Cytophagales; Microscillaceae | 2 | 0.98 | <0.001 |
| 3_Bin64 | Proteobacteria; Gammaproteobacteria; Betaproteobacteriales; **Burkholderiaceae**; Rubrivivax | 1-7 (4) | 2041_CU921717.1.1358 | 1072 | Proteobacteria; Gammaproteobacteria; Betaproteobacteriales; Burkholderiaceae | 1 | 0.96 | <0.001 |

a: rrn copy numbers from the rrnDB database correspond to taxa indicated in bold.

b: R and p values correspond to the correlation between MAGs and MA16s coverage across the 60 samples.
